# Flexural Behavior of Desert Sand Concrete-Filled Steel Tube: Experimental Validation, FEM Analysis, and Design Formulas

**DOI:** 10.3390/ma18102371

**Published:** 2025-05-20

**Authors:** Chao-Cheng Zhang, Fa-Xing Ding, Said Ikram Sadat, Fei Lyu, Xin-Yu Huang, Rui Gao, Tao Yu, Yu-Lin Liu

**Affiliations:** 1 School of Civil Engineering, Central South University, Changsha 410075, China; zjxrok002@163.com (C.-C.Z.); dinfaxin@csu.edu.cn (F.-X.D.); lyufei@csu.edu.cn (F.L.); 224811043@csu.edu.cn (X.-Y.H.); 2 Zhejiang Xinrui Construction Engineering Co., Ltd., Wenzhou 325000, China; 3 Engineering Technology Research Center for Prefabricated Construction Industrialization of Hunan Province, Changsha 410075, China; 4 China Railway Construction Engineering Group Co., Ltd., Beijing 100000, China; ztjgcsxzfxmb@163.com (R.G.); 18910618208@139.com (T.Y.); 15652961991@139.com (Y.-L.L.)

**Keywords:** concrete-filled steel tube (CFST), desert sand concrete, finite element analysis, flexural behavior, ultimate bending moment, design formulas

## Abstract

This study investigates the flexural performance of desert sand concrete-filled steel tube (DS-CFST) members through experimental validation and finite element modeling (FEM). An extensive database of square and circular CFST specimens subjected to pure bending was analyzed to validate an ABAQUS-based FEM. Parametric studies evaluated the influence of steel yield strength, steel ratio, stirrup confinement, and desert sand replacement ratio (r) on ultimate bending moment, stiffness, and failure modes. The results indicated that steel yield strength and section geometry significantly affected bending capacity, while desert sand substitution (r ≤ 1) had a negligible impact on capacity, reducing it by less than 3%. The FEM accurately predicted buckling patterns, moment-curvature relationships, and failure modes. New design formulas for predicting ultimate bending moment and flexural stiffness were proposed, demonstrating superior accuracy (mean error < 1%) compared to existing design codes (AIJ, AISC, GB). This study highlights that DS-CFST members, particularly circular sections, offer robust flexural performance, with enhanced ductility and uniform stress distribution. The findings underscore the potential of using desert sand as a sustainable material in concrete-filled steel tube structures without compromising structural integrity.

## 1. Introduction

Concrete, a globally used construction material, is primarily made with natural river sand. However, the extraction of river sand has raised significant environmental concerns, including resource depletion and ecological disruption [1,2]. This has led to an increasing need for sustainable alternatives. Desert sand (DS), which is abundantly available in arid regions, has emerged as a promising substitute for river sand in concrete production. DS offers an eco-friendly solution without substantially compromising the material properties of concrete.

Several studies have explored the potential of DS concrete as an alternative to traditional concrete mixes. Kazmi et al. [3] developed high-performance DS concrete using compression casting, which significantly enhanced compressive strength, durability, and resistance to chloride and carbonation ingress. Similarly, Akhtar et al. [4,5] reviewed the strength and durability characteristics of DS concrete, demonstrating that desert sand can replace up to 50% of river sand without negatively affecting the concrete’s performance. However, desert sand content exceeding this threshold may degrade strength and durability. Al-Harthy et al. [6] observed that desert sand improves concrete workability up to certain replacement levels, beyond which performance deteriorates. Moreover, Hamada et al. [7] and Gong et al. [8] demonstrated that treated desert sand improved the microstructure of the concrete, resulting in better freeze-thaw resistance and compressive strength.

In structural applications, DS concrete has gained attention for its potential to address the shortage of river sand. Li et al. [9] studied the cyclic behavior of reinforced concrete columns incorporating up to 60% DS and found that a replacement ratio between 20% and 40% enhanced seismic performance without compromising strength. Wang et al. [10] investigated dune sand concrete-filled steel tubular (CFST) columns and beams, showing that a 10% DS replacement resulted in good performance, with the steel tube providing effective confinement. Sadat et al. [11,12] developed design methods for rectangular and circular DS-CFST columns, highlighting that while higher DS replacement ratios reduce axial load-bearing capacity, the use of DS still yielded reliable results. Li et al. [13] found that DS concrete in short columns performed similarly to traditional concrete, with optimal performance at 40–60% DS replacement. Similarly, Li et al. [14,15,16] conducted studies on flexural and shear behaviors, demonstrating that DS replacement does not significantly affect failure modes, although ductility and energy dissipation improved with moderate DS content. These studies underscore that DS can effectively replace conventional sand in structural elements, particularly in regions with abundant desert sand.

Concrete-filled steel tube (CFST) composite structures, where concrete is used as the infill and steel serves as the structural casing, have become increasingly popular due to their enhanced structural performance. The composite action between the concrete and steel tube offers superior resistance to bending, axial compression, and seismic [17,18]. In particular, CFSTs are used in high-strength structural components such as columns, beams, and bridges. The addition of desert sand in the concrete mix for CFSTs could reduce the environmental footprint of construction projects, particularly in arid regions where desert sand is abundant. Several studies have investigated the behavior of CFSTs, with a focus on their flexural, axial, and seismic performance.

The flexural behavior of Concrete-Filled Steel Tubes (CFSTs) has been well-documented, highlighting the combined effect of concrete and steel in resisting bending. Ding et al. [19,20,21,22], Wang et al. [23], and Abed et al. [24] explored how different cross-sectional shapes, including circular and rectangular forms, influence the flexural capacity of CFSTs, particularly in recycled aggregate concrete (RAC). Further research on flexural behavior by Han [25] and Wang et al. [26] confirmed that CFSTs exhibit high flexural capacity and ductility due to the steel-concrete interaction, which allows for stress redistribution and greater overall performance under bending loads. Additionally, Montuori et al. [27,28] conducted experimental and finite element analyses on cyclic bending behavior in circular CFSTs, providing new insights into the structural performance, energy dissipation, and failure modes under cyclic loads. Their work emphasized the importance of accurately modeling the interaction between steel and concrete, which is critical for enhancing the safety and efficiency of CFST designs. However, studies specifically on the flexural behavior of desert sand concrete-filled steel tubes (DS-CFSTs) remain limited, despite the promising results of DS concrete in general.

This study aims to fill this gap by developing a finite element model (FEM) to analyze the flexural behavior of DS-CFST members, utilizing existing experimental data. The primary objectives of this study are as follows: (1) to establish a reliable FEM for simulating the moment-curvature response and failure modes of square and circular DS-CFSTs under pure bending conditions; (2) to assess the influence of key parameters such as the desert sand replacement ratio, steel ratio, and material strengths on flexural performance; (3) to develop new design equations for predicting ultimate bending moment and flexural stiffness, providing improved accuracy over existing design codes; and (4) to validate the FEM by comparing it with experimental results from previous studies, ensuring its reliability for parametric analysis and performance prediction of DS-CFST members.

## 2. Experimental Database

This study compiles test data for 136 concrete-filled steel tube (CFST) specimens 72 square and 64 circular from 18 different studies, as detailed in Table 1 and Table 2. The database includes information on section geometries (*D* = 33.66–456 mm, *t* = 1–14.6 mm), material properties (*f*_cu_ = 26.84–81.3 MPa, *f*_y_ = 235–460 MPa), and bending capacities (*M*_ue_, *M*_u,FE_).

## 3. Finite Element Modeling and Validation

### 3.1. Finite Element Model Establishment

#### 3.1.1. Element Type, Instruction, Mesh, and Boundary Conditions

**Figure 1:**
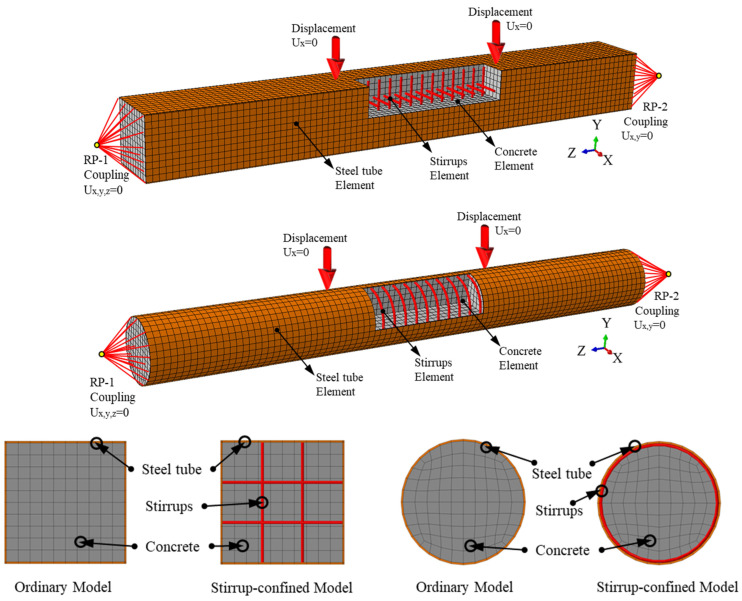
FE model under pure bending for square and circular sections.

The finite element model (FEM) for square and circular desert sand concrete-filled steel tube (DS-CFST) members under pure bending is developed using Abaqus 2020 Standard solver finite element (FE) software. The steel tube is simulated using a 4-node reduced integration shell element (S4R), while the DS concrete and cover plate are modeled with a three-dimensional 8-node reduced solid integration element (C3D8R). Stirrup reinforcement is modeled using truss elements (T3D2). The overall model uses structured meshing techniques, with a mesh size of *D*/10, as suggested by Ding et al. [44], to improve computational efficiency and accuracy. Schematic diagrams of the components are shown in Figure 1. Material and geometric nonlinearities are incorporated, and an incremental iterative method is employed to solve the model.

The interaction between the infilled DS concrete and the steel tube is modeled as surface-to-surface contact. Normal contact is defined as “hard contact”, and the Coulomb friction model is used to simulate the transmission of tangential forces. A friction coefficient of 0.5 is applied at the interface [19,22]. In the contact definition, the external surface of the DS concrete is set as the slave surface, and the inner surface of the steel tube is the master surface. The constraint between the DS concrete and the loading plate is modeled using a “Tie” constraint, while the Shell-to-Solid coupling constraint type is used between the steel tube and the loading plate. Stirrup elements are embedded within the concrete using the embedded method, and binding constraints are applied to ensure consistent deformation of the cover plate, steel tube, and core concrete. The stiffer cover plate is selected as the master surface, with the upper and lower surfaces of the steel tube concrete as the slave surfaces. The cover plate is modeled as a rigid plate with an elastic modulus of 1 × 10^12^ MPa and a Poisson’s ratio of 1 × 10^−7^.

The boundary conditions of the model are aligned with the experimental conditions, as illustrated in Figure 1. The model is subjected to four-point bending, which is consistent with the experimental setup. To simulate the simply supported boundary conditions, at support RP-1, in-plane rotation is released, allowing rotation but preventing displacement in the X, Y, and Z directions, ensuring the support behaves as a simple support. At support RP-2, both in-plane rotation and axial displacement are released along the specimen, permitting free rotation and longitudinal displacement, which simulates a simply supported beam. Considering that nonlinear calculations are more easily convergent under displacement loading, the load is applied to the specimen through the loading points via specified displacement.

#### 3.1.2. Material Constitutive Models

(1) Desert sand concrete

**Figure 2:**
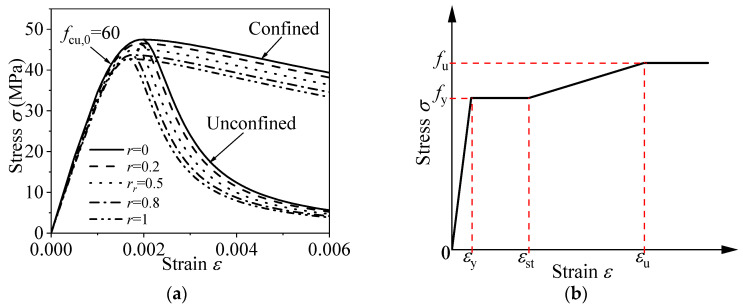
Uniaxial stress-strain curve: (**a**) Desert sand concrete with different replacement ratios; (**b**) Steel tube.

The uniaxial stress-strain constitutive relationship for DS concrete is based on the curve proposed by Sadat et al. [45]. The relationship is expressed as follows:

(1) $$y=\left\{\begin{array}{ll} \frac{A_{n\left(r\right)}x+(B_{n\left(r\right)}-1)x^{2}}{1+(A_{n\left(r\right)}-2)x+B_{n\left(r\right)}x^{2}} & x\leq 1\\ \frac{x}{\alpha_{n\left(r\right)}{\left(x-1\right)}^{2}+x} & x>1 \end{array}\right.$$

where, *A*_n(r)_ refers to the ratio of the elastic modulus of DS concrete to its peak secant modulus. *B*_n(r)_ determines the extent of attenuation in the ductility modulus during the ascending portion of the curve. The parameter *α*_n(r)_ describes the characteristics of the descending phase. The variables *f*_c(r)_ and *f*_t(r)_ represent the uniaxial compressive and tensile strengths of DS concrete, respectively, *r* is the DS replacement ratio. The specific values for these parameters are provided in Table 3. The DS concrete uniaxial stress-strain curve is illustrated in Figure 2a.

The triaxial constitutive model for DS concrete utilizes the plastic-damage model proposed by Ding et al. [46]. In the ABAQUS Concrete Damage Plasticity (CDP) model, the following triaxial plasticity parameters are used, as outlined in Table 4:

(2) Steel

The constitutive model for the steel components, including stirrups and steel tubes, is based on an elastic-plastic model proposed by Ding et al. [44] and is expressed as follows:

(2) $$\sigma =\left\{\begin{array}{ll} E_{s}\varepsilon & \varepsilon \leq \varepsilon_{y}\\ f_{y} & \varepsilon_{y}<\varepsilon \leq \varepsilon_{st}\\ f_{y}+E_{st}(\varepsilon -\varepsilon_{st})\; & \varepsilon_{st}<\varepsilon \leq \varepsilon_{u}\\ \;f_{u} & \varepsilon >\varepsilon_{u} \end{array}\right.$$

where *σ* and *ε* correspond to the stress and strain exhibited by the steel, respectively; *f*_y_ and *f*_u_ denotes the yield and ultimate strength of steel, respectively; *ε*_y_ signifies the yield strain, *ε*_st_ captures the strain at strengthening, *ε*_u_ denotes the strain at ultimate strength; *E*_s_ represents the elastic modulus of the steel, assumed to be 2.06 × 10^5^ MPa; and *E*_st_ represents the strengthened modulus of the steel, can be calculated as (*f*_u_−*f*_y_)/(*ε*_u_−*ε*_st_). *ε*_st_ = 0.02(if *f*_y_ ≤ 500 MPa) or *ε*_y_(if *f*_y_ > 500 MPa), *f*_u_/235 = 0.85 *f*_y_/235 + 0.72; *ε*_u/_*ε*_u,235_ = 1/1 + 0.15(*f*_y_/235−1)^1.85^. Figure 2b illustrates a schematic view of the adopted stress-strain relation for steel.

### 3.2. Finite Element Model Validation

To validate the finite element model, numerical simulations were conducted on pure bending tests of square and circular concrete-filled steel tubular (CFST) members, as reported in previous studies. The comparison between the FEM results and experimentally measured ultimate bending moments is presented in Table 1 for square sections and Table 2 for circular sections. For square sections, the average ratio of experimentally measured to FEM-calculated ultimate bending moments (*M*_ue_/*M*_u,FE_) is 1.026, with a coefficient of variation (CV) of 0.072. For circular sections, the average ratio is 1.033, with a CV of 0.076. These results demonstrate that the FEM accurately simulates the pure bending behavior of both square and circular CFST members, with low CV values indicating consistency and reliability in the predictions.

#### 3.2.1. Failure Mode

**Figure 3:**
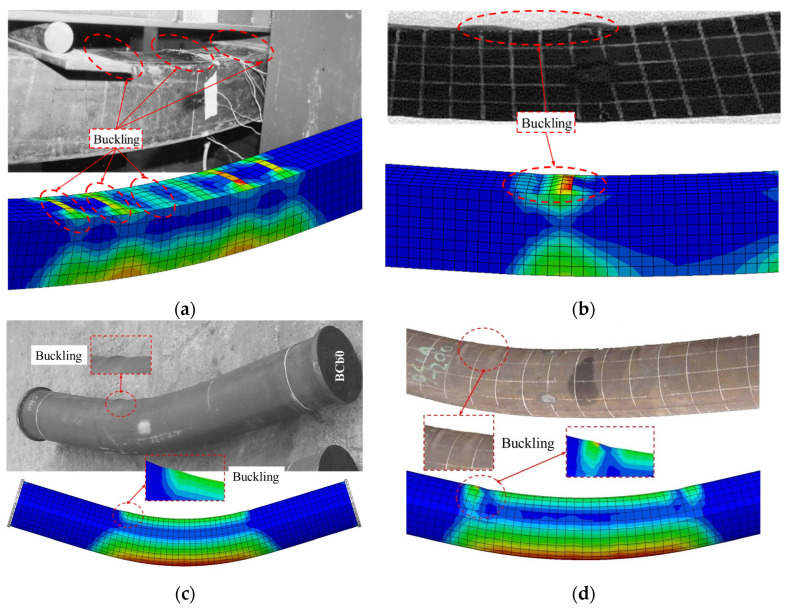
Comparison of experimental and FE failure mechanisms of specimens: Square (**a**) Brian Uy [30], (**b**) Han et al. [25]; Circular (**c**) Yang et al. [32], (**d**) Han et al. [31].

The comparison of failure modes between the FEM predictions and experimental results, shown in Figure 3, demonstrates strong agreement. For square sections, both methods reveal local buckling along the flat faces and corners, while circular sections exhibit uniform buckling around the circumference due to their geometry. The experimental failure modes were sourced from the existing database of literature, specifically from studies by Brian Uy [30] and Han et al. [25] for square sections, and Yang et al. [32] and Han et al. [31] for circular sections. The FEM results are from the present study. The FEM predictions align closely with the experimental observations, accurately capturing the buckling patterns and deformation zones. This confirms the reliability of the FEM in predicting the bending failure mechanisms of square and circular concrete-filled steel tube (CFST) members.

#### 3.2.2. Moment-Displacement and Moment-Curvature Curves

**Figure 4:**
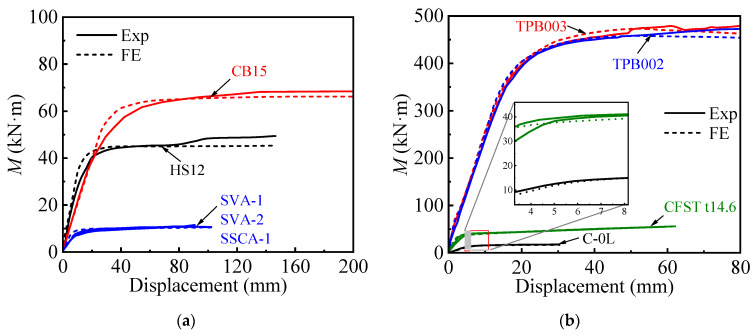
Comparison of experimental and FE-predicted moment-displacement curves: (**a**) Square; (**b**) Circular.

**Figure 5:**
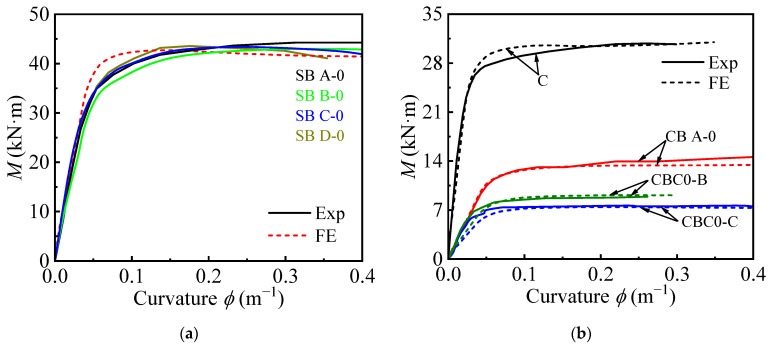
Comparison of experimental and FE-predicted moment-curvature curves: (**a**) Square; (**b**) Circular.

The comparison of measured and predicted moment-displacement and moment-curvature curves for circular and square CFST members, shown in Figure 4 and Figure 5, demonstrates strong agreement between the experimental results and FEM predictions. For both cross-sectional shapes, the FEM accurately captures the linear elastic stage, the transition to plastic behavior, and the ultimate bending moment. Circular sections exhibit smoother transitions and more uniform stress distributions, while square sections show slightly more pronounced stiffness degradation due to stress concentrations at the corners. Minor deviations in the post-peak behavior are attributed to material imperfections and localized effects not fully captured by the FEM model.

The midspan curvature (*ϕ*) is calculated from the maximum deflection (*u*_m_) using the equation:

(3) $$\phi =\frac{\pi^{2}}{L^{2}}u_{m}$$

where *L* is the column length. The curvature is derived from the second derivative of the displacement function, which is essential for understanding the moment-curvature relationship.

Overall, the FEM provides reliable predictions of moment-displacement and moment-curvature behavior, further validating its applicability for analyzing and designing CFST members under bending loads.

## 4. Finite Element Results and Analysis

### 4.1. Full Curve Analysis

**Figure 6:**
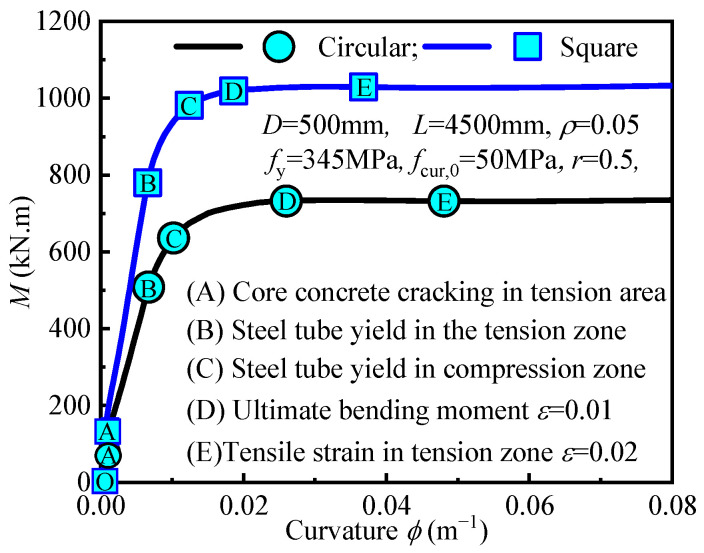
Moment-curvature curve of CFSTs under pure bending.

The bending moment-curvature curves for square and circular concrete-filled steel tube (CFST) sections under pure bending are shown in Figure 6. These curves show that both square and circular sections undergo three distinct stages before failure: the elastic stage (OB), the elastic-plastic stage (BD), and the plastic stage (DE). Key points include: Point A, where the concrete in the tension zone begins to crack; Points B and C, where the steel tube yields in the tension and compression zones, respectively; and Points D and E, where the strain in the steel tube in the tension zone reaches ε = 0.01 and ε = 0.02, respectively. During the elastic stage (OB), the bending moment increases rapidly with minimal curvature change. Concrete cracking at Point A slightly reduces stiffness, but the member remains elastic until the steel tube in the tension zone yields at Point B. The bond between the concrete and the steel tube limits crack propagation. In the elastic-plastic stage (BD), after yielding at Point B, deformation accelerates, and the compression zone steel yields at Point C. The yielding of the tension zone steel reduces stiffness, while the compression zone continues to provide support. As the load increases, the neutral axis shifts, and the yield zone expands. In the strain hardening stage (BC), curvature increases rapidly due to strain hardening and stress redistribution, while the bending moment increases at a slower rate. The curve flattens, indicating good ductility and the neutral axis stabilizes.

The uniform stress distribution in circular sections allows for delayed compression zone yielding, enhancing deformation capacity, while square sections achieve higher bending moments. Both sections exhibit excellent ductility during the plastic stage (DE), with stable moment-curvature behavior supported by strain hardening and stress redistribution.

### 4.2. Analysis of Factors Influencing Bending Moment and Flexural Stiffness

A total of 720 finite element models for pure bending cases were developed using the four-point loading method. The main parameters, as listed in Table 5, include material strengths (*f*_cu,r_ = 30 to 70 MPa; *f*_y_ = 245 to 420 MPa), steel ratio (*ρ* = 0.02 to 0.08), desert sand replacement ratio (*r* = 0 to 1), and equivalent stirrup ratios (*ρ*_sa_ = 0.0 to 0.245), for both square and circular sections.

#### 4.2.1. Material Strength

**Figure 7:**
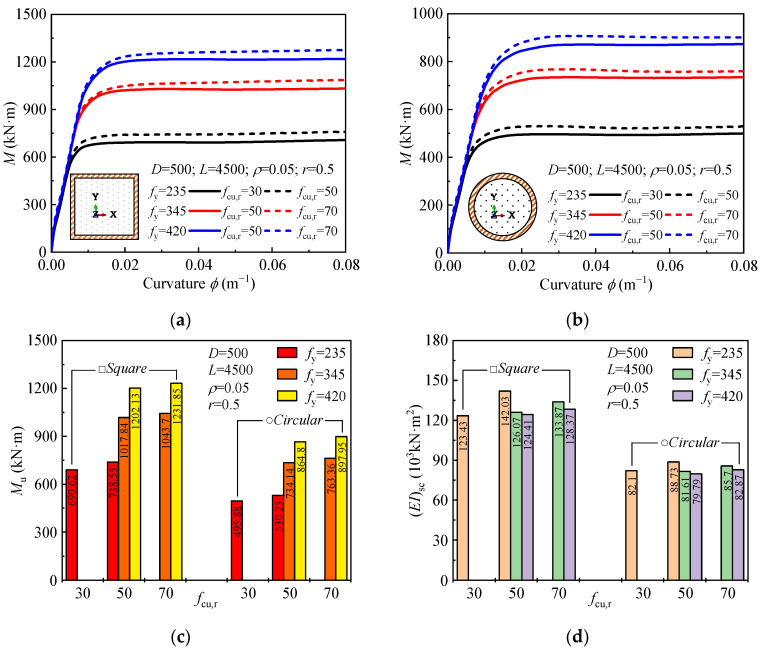
Effect of material strength on: (**a**,**b**) moment-curvature curves; (**c**) ultimate moment; (**d**) and flexural stiffness.

The influence of material strength on the moment-curvature curve, ultimate bending moment, and flexural stiffness is shown in Figure 7. Concrete strength (*f*_cu,r_) has a minimal effect on the ultimate bending moment, while steel strength plays a more significant role. As steel grade (*f*_y_) increases from 235 MPa to 345 MPa, the ultimate bending moment increases by approximately 37.9% for square sections and 38.5% for circular sections. Further increases in steel grade from 345 MPa to 420 MPa result in smaller increases in the moment (15.2% for square and 13.3% for circular sections). Regarding flexural stiffness, increasing concrete strength slightly improves stiffness, while higher steel strength reduces it. Numerically, the initial slope of the moment-curvature curve (from Figure 7a,b) decreases as the steel grade increases, indicating a reduction in the flexural stiffness. This reduction is due to the increased brittleness of higher-strength steel, which reduces the steel’s ability to deform and redistribute stresses, thus lowering the overall bending stiffness despite the increased moment capacity.

#### 4.2.2. Steel Ratio

**Figure 8:**
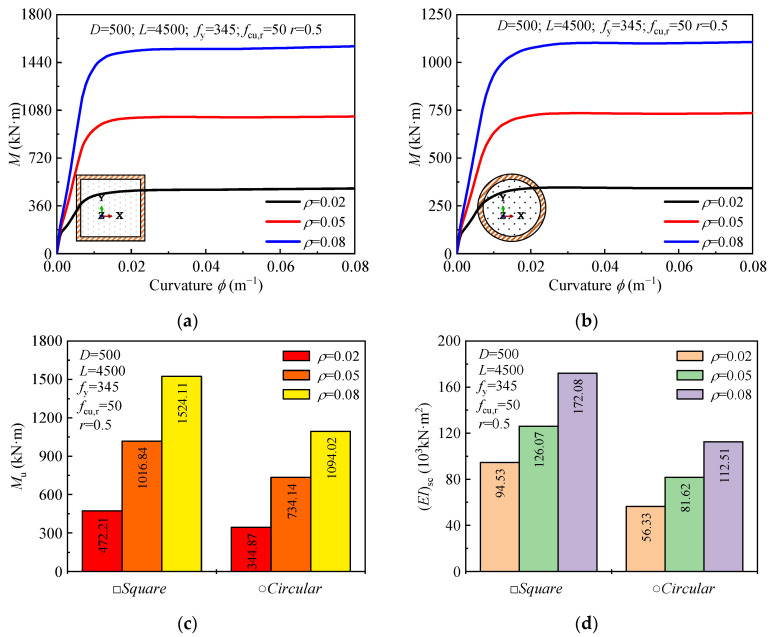
Effect of steel ratio (*ρ*) on: (**a**,**b**) moment-curvature curves; (**c**) ultimate moment; (**d**) and flexural stiffness.

The influence of the steel ratio (*ρ*) on the moment-curvature curve, ultimate bending moment, and flexural stiffness is presented in Figure 8. As the steel ratio increases from 0.02 to 0.05, the ultimate bending moment increases by 115.3% for square sections and 112.9% for circular sections. When the steel ratio is further increased from 0.05 to 0.08, the ultimate bending moment increases by 49% for both square and circular sections. Additionally, the flexural stiffness of the sections increases with the steel ratio. Specifically, as the steel ratio increases from 0.02 to 0.05 and 0.08, the flexural stiffness increases by 33.4% and 36.5% for square sections, and 44.9% and 37.9% for circular sections, respectively.

#### 4.2.3. Stirrup Confinement

**Figure 9:**
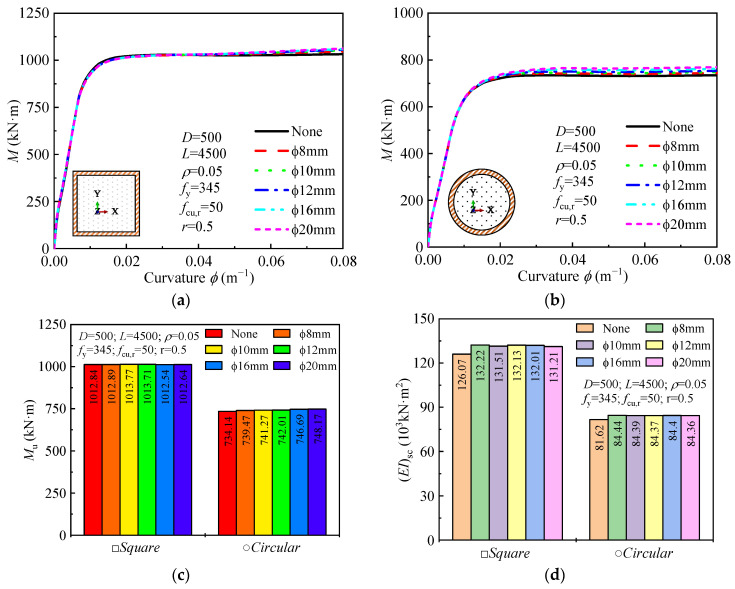
Effect of stirrup confinement on: (**a**,**b**) moment-curvature curves; (**c**) ultimate moment; (**d**) and flexural stiffness.

The influence of stirrup confinement on the moment-curvature curve, ultimate bending moment, and flexural stiffness is shown in Figure 9. The moment-curvature curves for different stirrup sizes closely overlap, indicating that stirrup confinement does not significantly alter the bending performance of the DS-CFST members. In the elastic region, the curves show a steep increase in bending moment with respect to curvature, which flattens out as the section moves into the plastic region, typical of CFST behavior. While there are minor variations in the plastic stage with increasing stirrup diameter (ϕ), these changes are minimal, suggesting that stirrup confinement has a small effect on the overall bending behavior. As the stirrup diameter increases from 8 mm to 20 mm, the ultimate bending moment and flexural stiffness increase by less than 5%. This minimal effect is consistent with findings from Ding et al. [19], who also reported a limited contribution of stirrup confinement to the bending performance of CFST columns.

#### 4.2.4. Desert Sand Replacement Ratio

**Figure 10:**
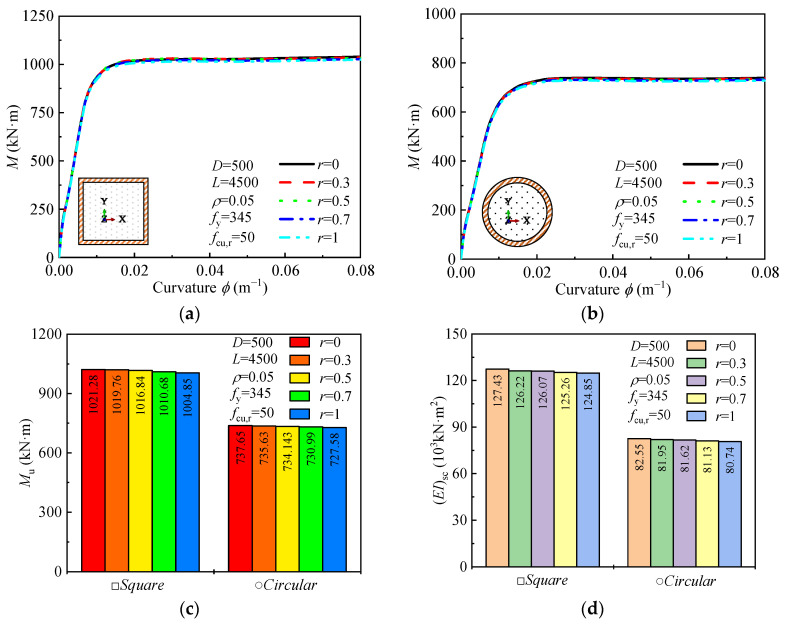
Effect of *r* on: (**a**,**b**) moment-curvature curves; (**c**) ultimate moment; (**d**) and flexural stiffness.

The effect of the desert sand replacement ratio (*r*) on the moment-curvature relationship, ultimate bending moment, and flexural stiffness of square and circular DS-CFST sections is shown in Figure 10. As r increases from 0 to 1, the ultimate bending moment and flexural stiffness of the sections decrease by less than 3%. This indicates that desert sand replacement has a negligible impact on the bending performance of both square and circular sections. The minimal reduction in bending moment and stiffness suggests that the substitution of desert sand does not significantly affect the structural capacity of these sections under bending. These results imply that dune sand, when used as a partial replacement in concrete, does not compromise the bending strength or stiffness of the sections, making it a viable option for sustainable construction applications without detrimental effects on performance.

### 4.3. Failure Analysis

**Figure 11:**
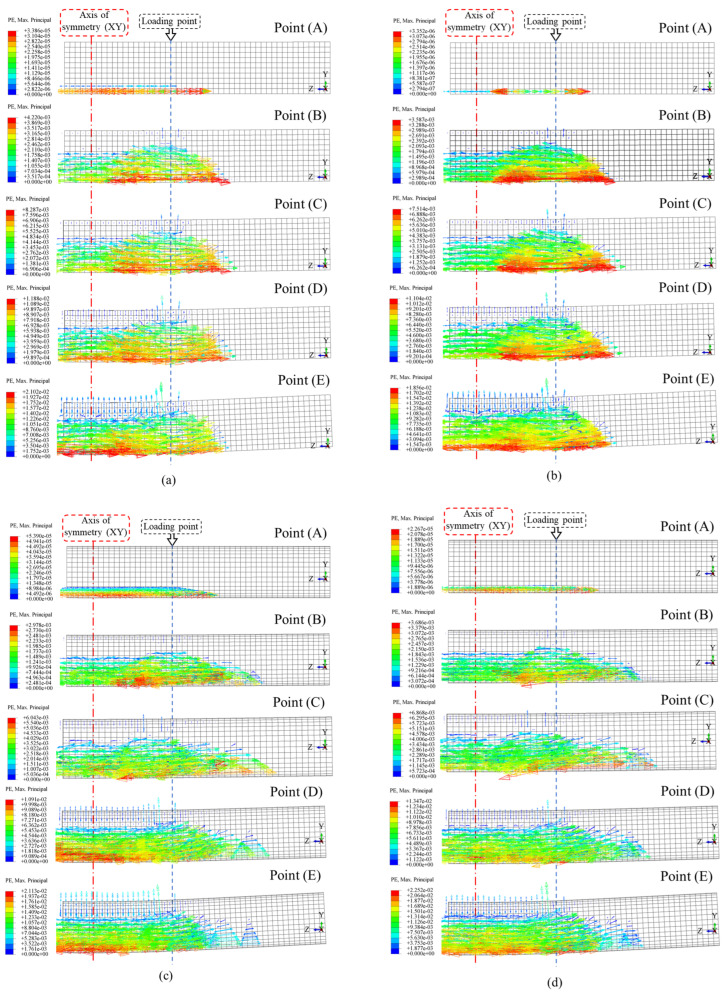
Development law of plastic strain in concrete: (**a**) square ordinary (*ρ*_sa_ = 0); (**b**) square stirrup-confined (*ρ*_sa_ = 0.0245); (**c**) circular ordinary (*ρ*_sa_ = 0); (**d**) and circular stirrup-confined (*ρ*_sa_ = 0.0245).

The crack development in square and circular concrete-filled steel tubes (CFSTs) under pure bending was analyzed using the ABAQUS 2020 software. The results are illustrated in Figure 11. Vector symbols were used to represent concrete cracking, with the direction of the arrows indicating plastic strain direction, and the density of the vectors reflecting crack severity. The plastic strain evolution was studied for four configurations: square ordinary (*ρ*_sa_ = 0), square stirrup-confined (*ρ*_sa_ = 0.0245), circular ordinary (*ρ*_sa_ = 0), and circular stirrup-confined (*ρ*_sa_ = 0.0245). At the early loading stage (Point A), plastic strain initiates symmetrically near the loading point, with ordinary models exhibiting lower crack severity compared to stirrup-confined models. As the tensile steel yields (Point B), cracks propagate beneath the neutral axis. Stirrup-confined models exhibit better strain distribution and reduced crack localization. At Point C, compression zone yielding becomes evident, with square sections showing more localized strain due to corner stress concentrations, while circular sections exhibit smoother strain gradients. By the ultimate strain state (Point D, *ε* = 0.01), ordinary models experience dense strain localization, whereas stirrup-confined models demonstrate a more uniform strain distribution. At the post-ultimate stage (Point E, *ε* = 0.02), severe strain localizes under the loading point in ordinary models, while confinement in stirrup-confined models delays failure and enhances crack resistance. These results confirm that circular sections achieve broader, smoother strain distributions due to their uniform geometry, while square sections exhibit higher stress concentrations at the corners. Stirrup confinement significantly mitigates crack propagation, improves strain distribution, and enhances the structural performance of CFSTs under pure bending.

**Figure 12:**
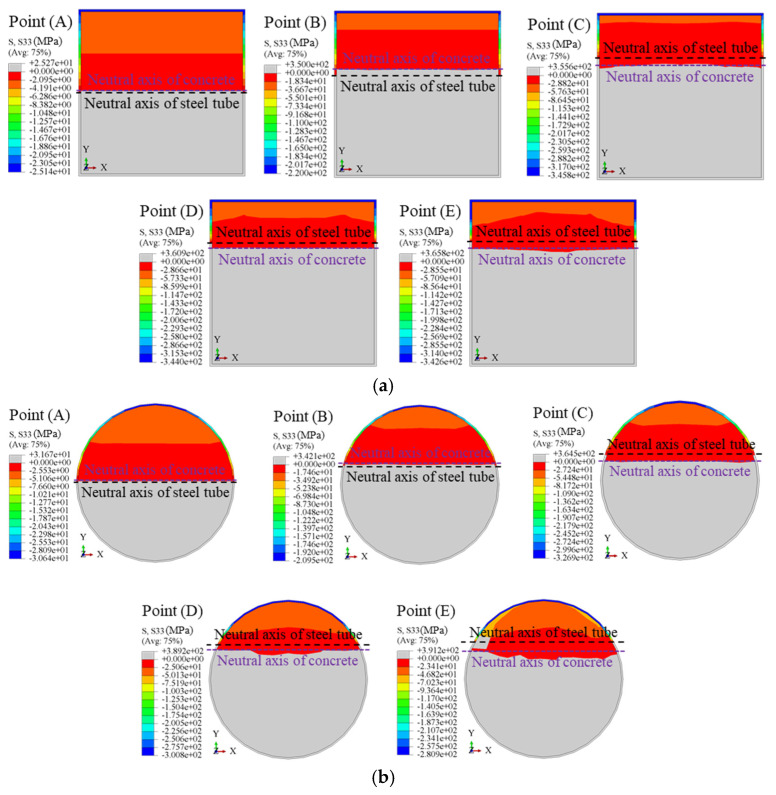
Variation law of neutral axis between mid-span steel tube and concrete during the loading process: (**a**) square section; (**b**) circular section.

Figure 12 illustrates the variation in the neutral axis of the steel tube and concrete during the loading process. Initially, the neutral axis coincides with the centroidal axis, indicating a uniform stress distribution. As the load increases, the neutral axis of the concrete shifts upward due to cracking in the tension zone, while the steel tube maintains a lower neutral axis, reflecting relative slip. In square sections, the neutral axis shifts abruptly at higher loads due to stress concentrations at the corners and compression zone yielding. In contrast, circular sections exhibit smoother and more uniform shifts due to their superior confinement and stress distribution. At the ultimate load (*ε* = 0.01), the neutral axis stabilizes, and by the post-ultimate stage (*ε* = 0.02), maximum slip occurs. Square sections show localized strain, while circular sections maintain broader, uniform deformation.

**Figure 13:**
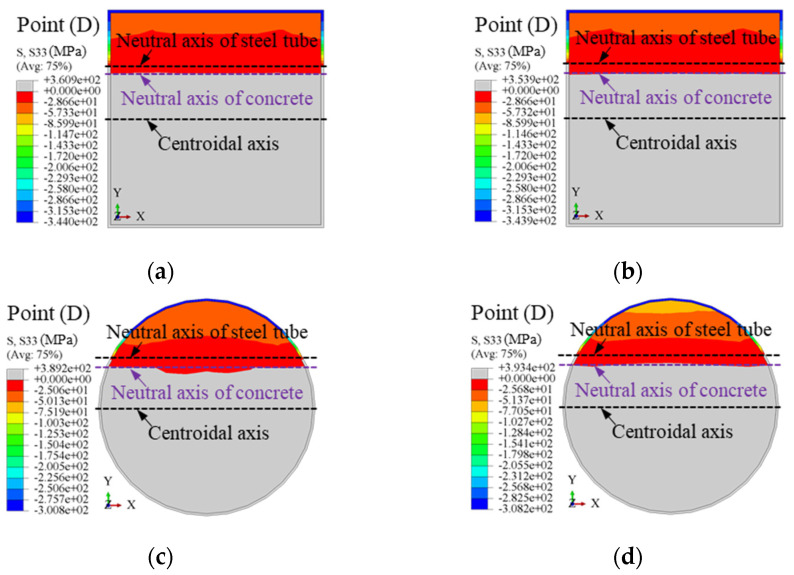
Comparison of longitudinal stress contours at mid-span section: (**a**) square ordinary (*ρ*_sa_ = 0); (**b**) square stirrup-confined (*ρ*_sa_ = 0.0245); (**c**) circular ordinary (*ρ*_sa_ = 0); (**d**) and circular stirrup-confined (*ρ*_sa_ = 0.0245).

Taking square and circular CFSTs with dimensions *D* = 500 mm, member length *L* = 4500 mm, concrete compressive strength *f*_cu,r_ = 50 MPa, steel yield strength *f_y_* = 345 MPa, steel ratio *ρ* = 0.05, and stirrup ratio *ρ*_sa_ = 0.0245 as examples, the longitudinal stress contours at the mid-span section under pure bending at the ultimate bending moment (Point D) are shown in Figure 13. The gray areas indicate tension zones, while darker colors represent higher compressive stresses. The following observations are made: (1) Square Sections: The neutral axis of the concrete is closer to the centroidal axis than that of the steel tube, indicating the concrete’s primary role in resisting compressive stresses. Stress distribution in square sections is less uniform due to geometric stress concentrations at the corners. The stirrup-confined square section (*ρ*_sa_ = 0.0245) shows no significant change in stress distribution compared to the ordinary square section (*ρ*_sa_ = 0), indicating that stirrup confinement has minimal influence on bending performance in square sections (see Figure 13a,b). (2) Circular Sections: The neutral axis of the concrete is closer to the centroidal axis than that of the steel tube, reflecting more balanced stress sharing. Circular sections demonstrate better stress distribution and reduced stress gradients due to their symmetric geometry. Stirrup confinement (*ρ*_sa_ = 0.0245) slightly improves stress uniformity, but the improvement is marginal, as the ordinary circular model (*ρ*_sa_ = 0) already exhibits effective confinement and stress distribution (see Figure 13c,d). (3) Stirrup Confinement: Across both square and circular sections, stirrup confinement has a negligible impact on stress distribution under pure bending, as evidenced by the minimal numerical differences in stress contours. This suggests that section geometry plays a more significant role in stress sharing than stirrup confinement.

### 4.4. Stress Distribution Analysis at Different Loading Stages

**Figure 14:**
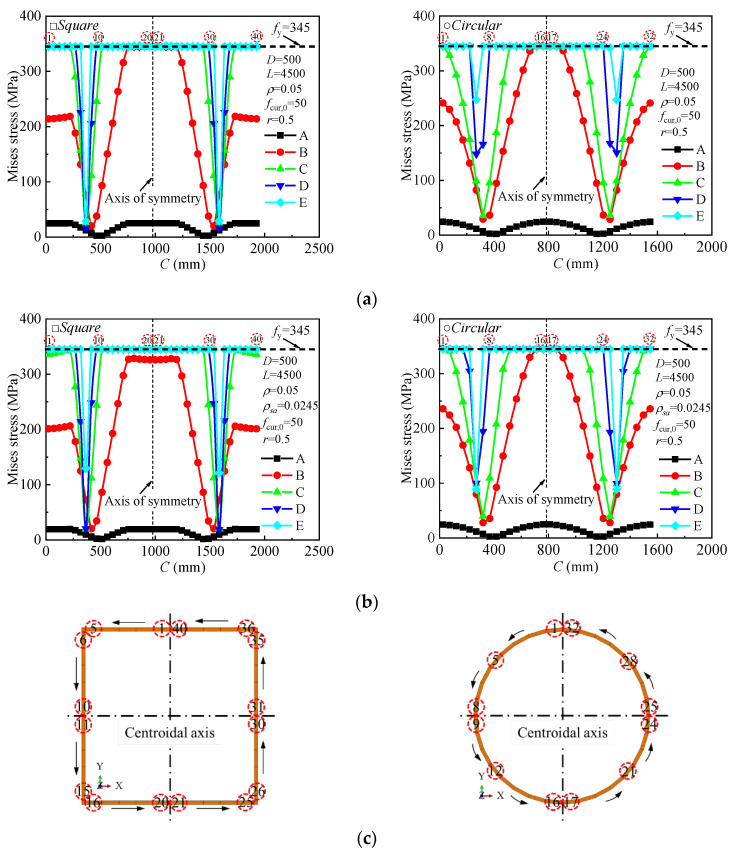
Development of von Mises stress along the perimeter of the steel tube at different loading stages: (**a**) ordinary model; (**b**) stirrup-confined model; (**c**) marked points on the steel tube perimeter for stress analysis.

The development of von Mises stress at the mid-span section of the steel tube at various loading stages is shown in Figure 14. The following observations can be made: (a) For the ordinary square model, as the load increases, the steel tube in the tension zone (points 16–25) yields first, followed by yielding in the compression zone (points 1–15, 26–40). In the stirrup-confined model, yielding occurs first below the centroidal axis (points 11–30), followed by yielding above the centroidal axis (points 1–10, 31–40). The yield zone progressively expands toward the neutral axis, with the yield strength being gradually reached, as seen at points 8–9 and 32–33. (b) For the ordinary circular model, as the load increases, the steel tube in the tension zone (points 15–18) yields first, followed by yielding in the compression zone (top). The yield region then extends from both the tension and compression zones toward the neutral axis, enlarging progressively. A similar trend is observed in the stirrup-confined model. (c) The neutral axis gradually shifts upward as the specimen deforms, while the steel tubes near the neutral axis remain unyielded throughout the loading process. This indicates that the steel tube near the neutral axis is effectively resisting deformation, contributing to the composite action of the CFST. (d) The addition of stirrups and the section type (square or circular) have little effect on the overall evolution of the von Mises stress distribution. The trends of stress development remain consistent across both ordinary and stirrup-confined models. (e) As loading progresses, the compression zone near the neutral axis develops, but the steel tube near this zone remains unyielded, suggesting that the steel tube effectively resists compressive stresses and maintains structural integrity throughout the loading process.

**Figure 15:**
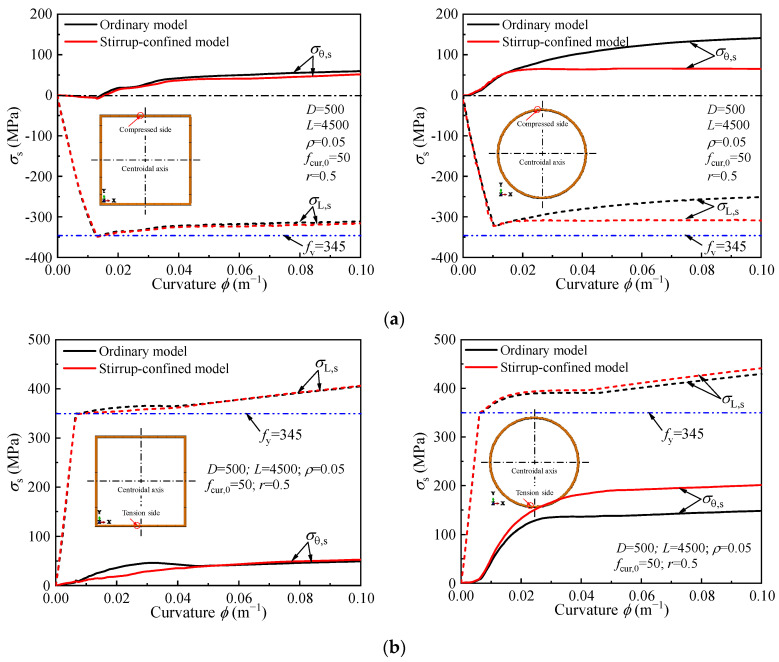
Effect of stirrup confinement on the stress-curvature curve of the steel tube: (**a**) Compression Side; (**b**) Tension side.

The effect of stirrup confinement on the steel tube stress in concrete-filled steel tubes (CFSTs) under pure bending is shown in Figure 15. In both the ordinary and stirrup-confined models, the steel tube in the tension zone yields first, while the compression zone steel tube does not reach its yield point, particularly in the circular section. Stirrup confinement results in a slight increase in hoop stress (*σ*_θ,s_) but has minimal effect on the longitudinal stress (*σ*_L,s_) distribution within the steel tube. In both cases, the stress behavior and yielding in the steel tube are similar, with no significant changes due to stirrup confinement. Additionally, stirrup confinement does not significantly enhance the ultimate bending moment capacity of the CFST specimens. Overall, the influence of stirrup confinement on the steel tube’s behavior under pure bending is limited, with the circular section showing no compression zone yielding throughout the loading stages.

**Figure 16:**
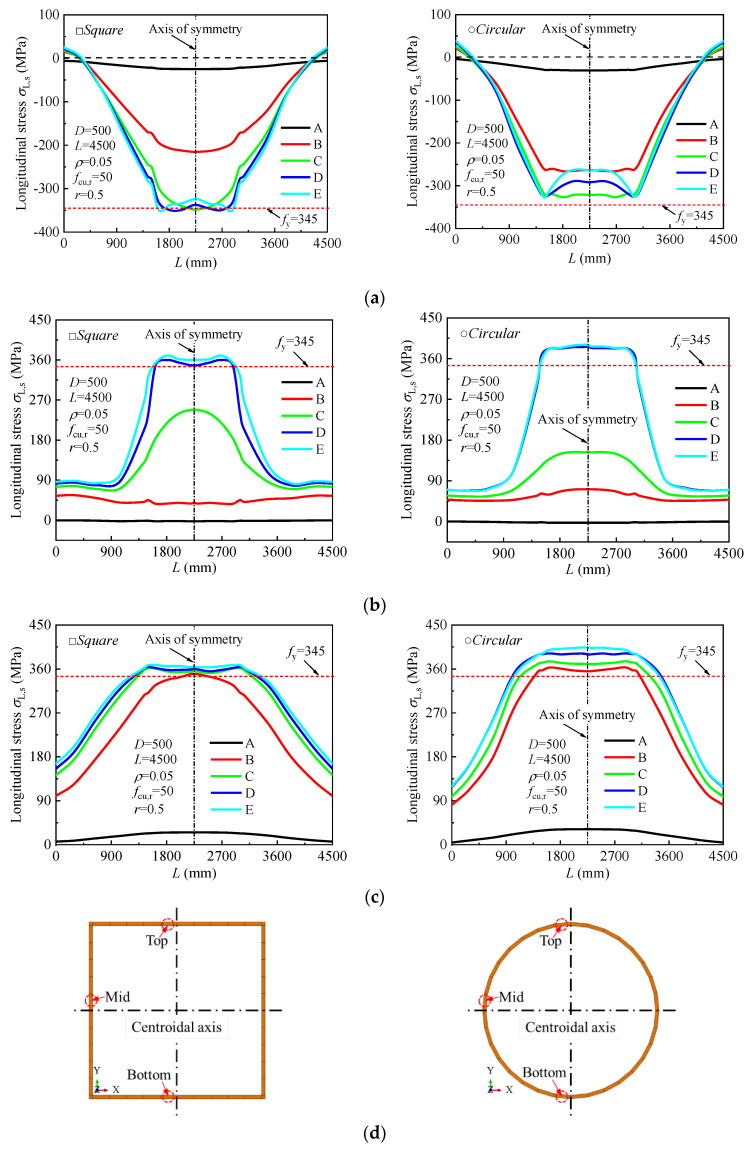
Development of the longitudinal stress in the steel tube along the length of the specimen at different loading stages: (**a**) Top; (**b**) Mid; (**c**) Bottom; (**d**) Marked points along the steel tube for stress analysis.

Figure 16 illustrates the development of longitudinal stresses along the full length of the specimen under pure bending. As shown in Figure 16a, the entire top surface of the steel tube is under compressive stress, with compressive stresses in the pure bending region (*l*_0_) being higher than those in adjacent regions. This indicates that, under pure bending, the top portion of the tube experiences significant compressive forces. Figure 16b,c show that the middle and bottom portions of the steel tube are under tensile stress, with the tensile stress in the pure bending region being greater than in other regions. The tensile stresses increase progressively as the load is applied.

**Figure 17:**
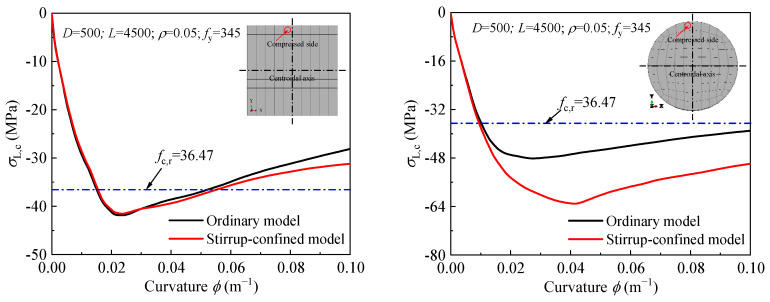
Effect of stirrup confinement on the stress-curvature curve of the concrete.

Figure 17 shows the longitudinal stress distribution in the top compression zone of the concrete for both square and circular CFST specimens under pure bending. In the square section, the concrete in the compression zone experiences increasing compressive stress as the load increases, with the stress in this region consistently higher than in other parts of the section, highlighting the concrete’s primary role in resisting compressive forces. The stress distribution is non-uniform, with concentrations near the corners. In contrast, the circular section shows a more uniform stress distribution in the top compression zone, with compressive stress also increasing with the load but more evenly spread due to the section’s symmetric geometry, resulting in less localized stress. In the ordinary models, the maximum compressive stress reaches 41.87 MPa for square sections and 48.12 MPa for circular sections, which is 14.82% and 32% higher than the axial compressive strength (*f*_c,0.5_ = 36.47 MPa), respectively. In models with stirrup confinement, the maximum compressive stress remains unchanged at 41.88 MPa for square sections, while it increases to 63.1 MPa for circular sections. These results indicate that stirrup confinement has no effect on improving the concrete’s compressive strength in square sections but provides a marginal improvement in circular sections.

**Figure 18:**
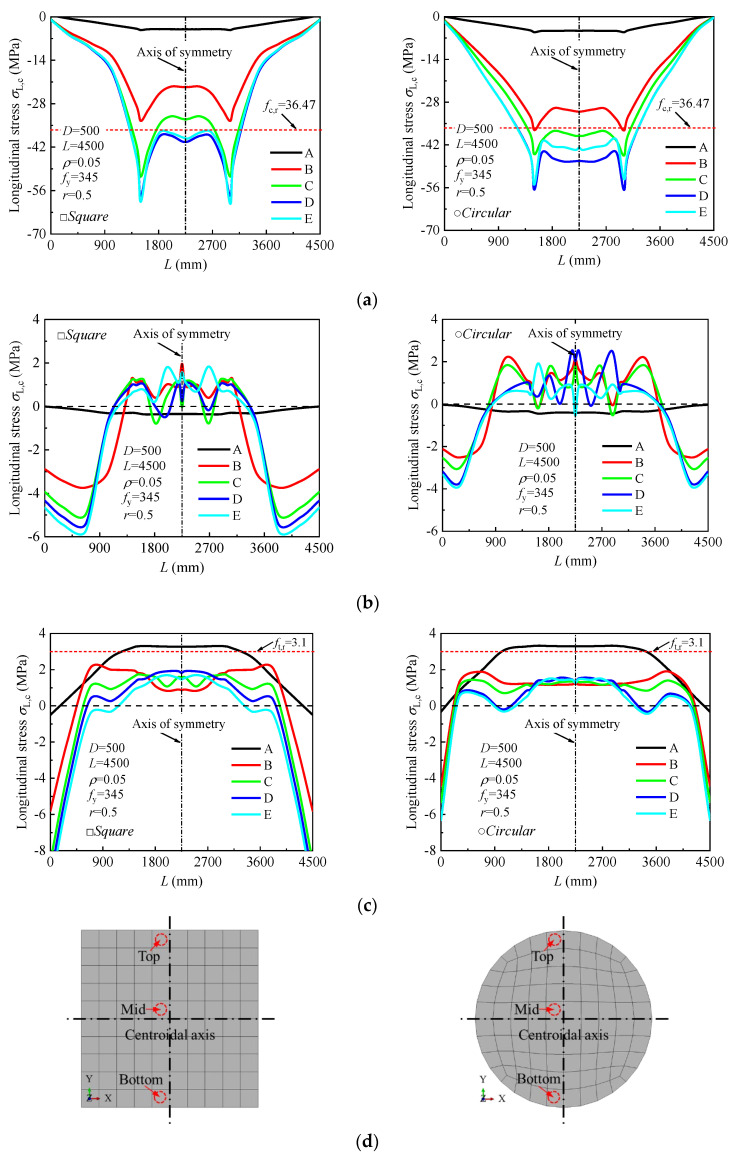
Development of the longitudinal stress in the concrete along the length of the specimen at different loading stages: (**a**) Top; (**b**) Mid; (**c**) Bottom; (**d**) Marked points along the concrete core for stress analysis.

Figure 18 illustrates the variation of longitudinal stress in the core concrete along the full length of the specimen under pure bending. As shown in the figure, the top portion of the core concrete remains in compression throughout the loading stages. The middle and bottom sections of the core concrete are subjected to tension, resulting in cracking in the pure bending region and part of the bending-shear region. Near the supports, the concrete is primarily in compression, but the compressive stress is relatively low, indicating that the concrete in these regions contributes minimally to resisting bending. The tension zone, where cracking occurs, indicates the development of plastic deformation in the concrete due to the applied loads. Overall, the stress distribution in the core concrete shows a typical bending response, with the upper region primarily in compression and the lower region experiencing tensile stresses, leading to localized cracking.

## 5. Prediction Design Formulas

### 5.1. Ultimate Bending Moment

The ultimate moment of the circular and square CFST pure bending members is calculated using the following formula:

(4) $$M_{u}=\gamma_{m}W_{\mathrm{sc}}f_{\mathrm{sc},u}$$

In the equation, γ_m_ represents the flexural stiffness coefficient of the section, *W*_sc_ is the flexural modulus of the section, and *f*_sc,u_ is the ultimate strength. The ultimate strength is defined as *f*_sc,u_ = *N*_u_/*A*_sc_, *A*_sc_ = *A*_c_ + *A*_s_. Here, *N*_u_ is derived from Equation (5) for the square section [11] and Equation (6) for the circular section [12], and *A*_sc_ is the cross-sectional area of the CFST, with *A*_c_ representing the concrete area and *A*_s_ representing the steel area.

(5) $$\left\{\begin{array}{l} N_{u}=f_{c}A_{c}+k_{2}f_{s}A_{s}\\ k_{1}=1.04-0.06\ln(B/D-0.9) \end{array}\right.$$

(6) $$\left\{\begin{array}{l} N_{u}=f_{c}A_{c}+k_{2}f_{y}A_{s}\\ k_{2}=1.58 \end{array}\right.$$

The bending modulus *W*_sc_ for circular section and square section is derived from the following formula:

(7) $$\left\{\begin{array}{l} W_{Sc}^{Cir}=\frac{\pi D^{3}}{32}\\ W_{Sc}^{Squ}=\frac{D^{3}}{6} \end{array}\right.$$

**Figure 19:**
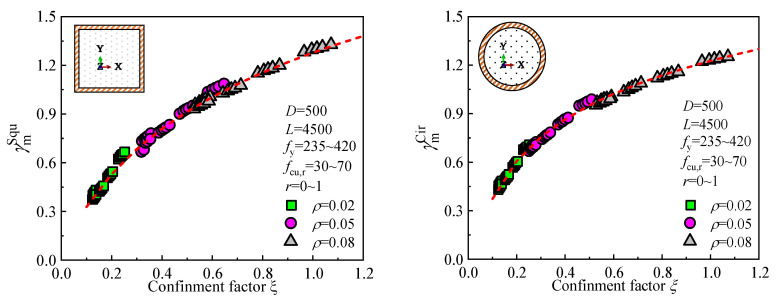
Fitted curve of the section flexural stiffness coefficient (*γ*_m_) vs. confinement coefficient (*ξ*).

Figure 19 illustrates the relationship between the section’s flexural stiffness coefficient *γ*_m_ and the confinement coefficient *ξ*, based on numerous numerical analyses. Through numerical fitting, the following equations can be obtained:

(8) $$\mathrm{Square}\;\mathrm{section}\;\;\;\;\;\gamma_{m}^{Squ}=1.179+0.658\ln(\xi +0.178)$$

(9) $$\mathrm{Circular}\;\mathrm{section}\;\;\;\;\;\gamma_{m}^{Cir}=1.213+0.415\ln(\xi +0.032)$$

**Figure 20:**
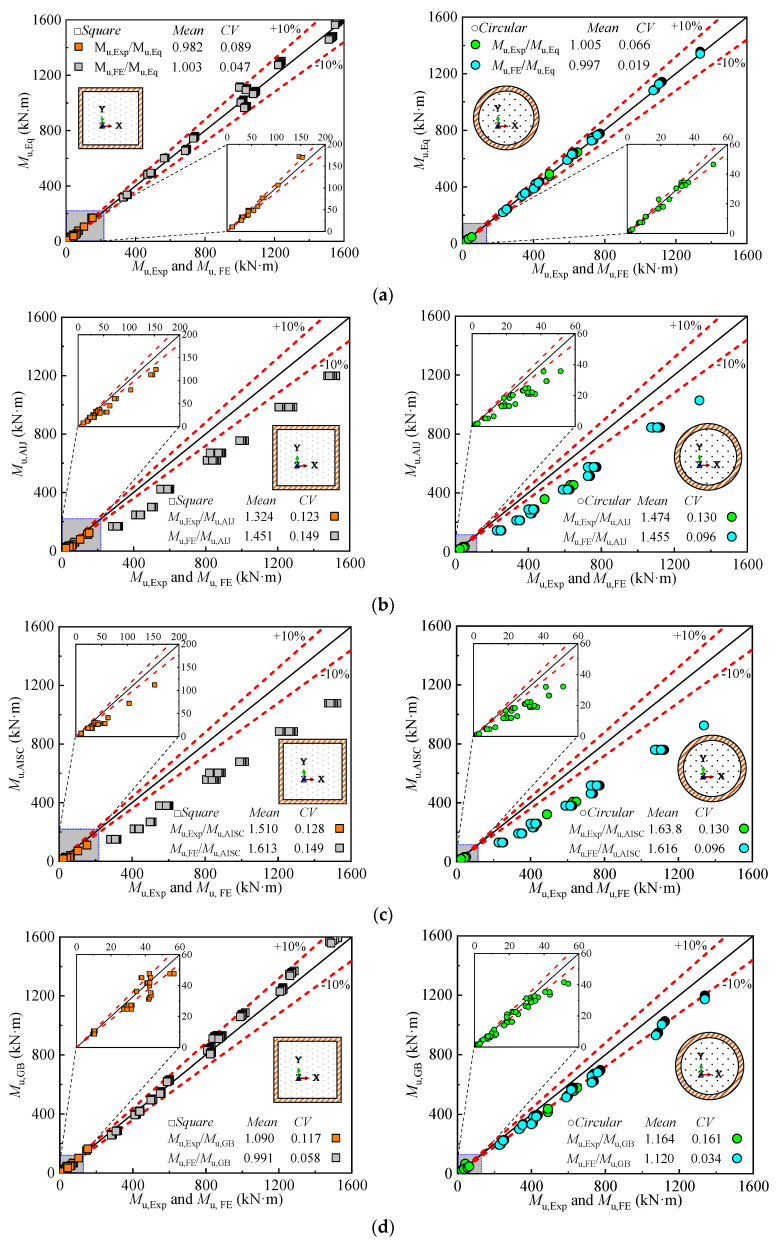
Comparison of the ultimate moment proposed formula and codes formula calculation results with experimental and finite element results: (**a**) The proposed equation; (**b**) AIJ; (**c**) AISC-LFRD; (**d**) GB50936-2014.

To assess the accuracy of the proposed formulas, the ultimate bending moments of square and circular DS-CFST sections were compared with experimental results (*M*_u,Ex_), finite element (FE) results (*M*_u,FE_), and the results from various international calculation formulas, as presented in Table 6 and Figure 20. The experimental and FE results were used as benchmarks, while the calculated bending moments were derived using the proposed formula and the formulas from international codes (*M*_u,Eq_). The following observations can be made:

For Square Sections: The ratios of experimental and FE to calculated bending moments (*M*_u,Ex_, *M*_u,FE_/*M*_u,Eq_) for the AIJ, AISC, and GB codes were 1.388, 1.562, and 1.041, respectively. The coefficients of variation (CV) for these values were 0.136, 0.139, and 0.088. These results indicate that the AIJ and AISC codes significantly underestimate the bending moment capacity of the sections, as they do not account for the confinement effect between concrete and steel, thus neglecting the contribution of concrete to the bending capacity. On the other hand, the GB specification provides more accurate predictions, with a calculated value of 1.041 and a CV of 0.088, making it closer to the experimental and FE results.

For Circular Sections: The ratios of *M*_u,Ex_, *M*_u,FE_/*M*_u,Eq_ for the AIJ [47], AISC [48], and GB50936-2014 [49] codes were 1.465, 1.627, and 1.142, respectively, with CVs of 0.113, 0.113, and 0.098. Similar to the square sections, the AIJ and AISC codes underestimate the bending moment, again due to the neglect of the concrete-steel confinement effect. The GB code, however, provides a better approximation, with a calculated value of 1.142 and a CV of 0.098.

Comparison with International Codes: When comparing the proposed formula with the international codes, the results indicate that the proposed formula yields bending moment values that are very close to the experimental and FE results for both square and circular sections. For square sections, the mean value of *M*_u,Ex_, *M*_u,FE_/*M*_u,Eq_ is 0.993, with a CV of 0.068, and for circular sections, the mean value is 1.001, with a CV of 0.043. These results show that the proposed formula provides a reliable and accurate prediction for both types of sections, with low variability, indicating its robustness in capturing the bending capacity of DS-CFST sections.

### 5.2. Flexural Stiffness

Currently, design codes and regulations provide calculation methods and formulas for determining the bending stiffness of different section steel tube concrete (CFST) members. These formulas consider the influence of concrete cracking on bending stiffness to varying degrees. The formula for calculating the flexural stiffness of CFST is as follows:

(10) $${\left(EI\right)}_{\mathrm{sc}}=E_{s}I_{s}+k_{z}E_{c}I_{c}$$

In this formula, (*EI*)_sc_ represents the combined flexural stiffness of the DS-CFST, *E*_s,_ and *E*_c_ are the elastic moduli of the steel tube and concrete, respectively, and *I*_s_ and *I*_c_ are the section moments of inertia of the steel tube and concrete.

The coefficient *k*_z_ is a reduction factor for the flexural stiffness of the concrete, accounting for cracking. The initial flexural stiffness of the DS-CFST is determined by the secant slope corresponding to a bending moment of 0.4 times the ultimate bending moment (*M* = 0.4 *M*_u_) [20] in the elastic phase of the moment-curvature curve.

The expression for the flexural stiffness reduction factor *k*_z_ is obtained by fitting the results of finite element models for 720 sets of square and circular DS-CFST pure bending components, as analyzed in Section 4. The expressions are as follows:

(11) $$k_{z}^{\mathrm{Squ}}=0.33+0.64\rho$$

(12) $$k_{z}^{\mathrm{Cir}}=0.30-0.49\rho$$

This coefficient takes into account the influence of parameters such as concrete strength grade, steel yield strength grade, and section steel ratio.

**Figure 21:**
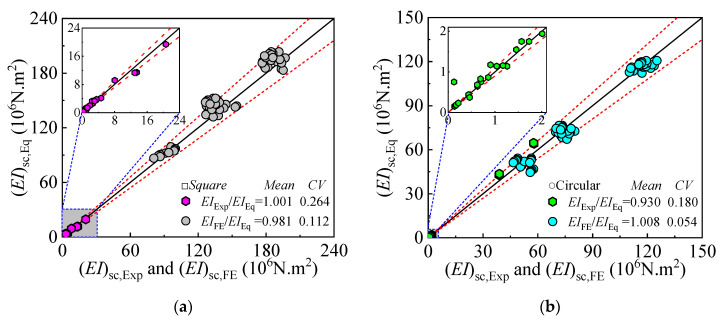
Comparison between the flexural stiffness calculation values from the formula, FE analysis, and experimental results for pure bending components of DS-CFST: (**a**) Square, (**b**) Circular.

The comparison between the flexural stiffness calculation results from the formula, finite element analysis, and experimental data is shown in Figure 21. It can be observed that the average values of (*EI*_Exp_/*EI*_Eq_) and (*EI*_FE_/*EI*_Eq_) are 1.001 and 0.981 for square sections, and 0.93 and 1.008 for circular sections, with coefficients of variation (CV) of 0.264 and 0.112 for square sections, and 0.18 and 0.054 for circular sections, respectively. These results demonstrate good agreement between the experimental values, finite element analysis results, and formula-calculated values, indicating the validity of the proposed stiffness reduction factor for DS-CFST members.

## 6. Conclusions

This study investigates the flexural behavior of desert sand concrete-filled steel tube (DS-CFST) members through experimental validation and finite element modeling. Based on the analysis, the following conclusions are drawn:

1. The ABAQUS-based finite element model accurately predicted the failure modes and moment-curvature relationships for both square and circular sections. The model demonstrated strong agreement with experimental results, successfully capturing the three-stage bending response.
2. Steel yield strength significantly influenced the ultimate bending moment, with increases of up to 38.5% as the steel yield strength rose from 235 MPa to 420 MPa. The steel ratio also played a crucial role, with a 115% and 113% increase in moment capacity for square and circular sections, respectively. While stirrup confinement had minimal effect on bending capacity, it helped control crack propagation. Desert sand replacement had a negligible impact on capacity, confirming its feasibility as a sustainable material for concrete production.
3. Square sections exhibited localized buckling due to stress concentrations at the corners, whereas circular sections showed more uniform stress distribution and greater ductility. The neutral axis shifted more abruptly in square sections compared to the smoother transition observed in circular sections.
4. New formulas for predicting ultimate bending moment and flexural stiffness were developed, incorporating confinement effects and stiffness reduction factors. These formulas demonstrated superior accuracy compared to existing design codes (AIJ, AISC, GB50936-2014), with low coefficients of variation, thus enhancing the reliability of DS-CFST design.
5. This research provides valuable tools for designing DS-CFST members, highlighting the potential of desert sand as a sustainable construction material. Future research could explore long-term durability, cyclic loading performance, and the optimization of stirrup confinement strategies to further enhance seismic resistance and overall performance.

## Figures and Tables

**Table 1:** Specimen details and comparison of bending moment test results with FEM results for square CFSTs.

Specimen Label	Ref.	*D*/mm	*t*/mm	*l*_0_/mm	*a*/mm	*f*_cu_/MPa	*f*_y_/MPa	*M*_ue_/kN·m	*M*_u,FE_/kN·m	*M*_ue_/*M*_u,FE_
CB12	[29]	152	4.8	1975	235	58.8	389	73.6	71.52	1.03
CB13		152	4.8	8430	463	53.5	389	75.1	72.01	1.04
CB15		152	4.8	3040	768	51.5	389	71.3	72.90	0.98
CB22		152	9.5	1976	236	58.6	432	146.5	119.51	1.23
HS6	[30]	126	3	1800	600	62.5	300	27.9	27.99	1.00
HS12		126	3	2250	750	62.5	300	42.4	44.53	0.95
NS6		186	3	2700	900	40.0	300	62.6	56.64	1.11
NS12		246	3	3600	1200	47.5	300	103.5	105.23	0.98
NS18		306	3	4500	1500	47.5	300	153	155.38	0.98
RB1-1	[25]	120	3.84	1000	250	27.3	330.1	29.34	28.45	1.03
RB2-1		120	3.84	1000	250	35.2	330.1	30.16	29.06	1.04
RB2-2		120	3.84	1000	250	35.2	330.1	32.25	29.06	1.11
RB2-3		120	3.84	1000	250	35.2	330.1	31.69	29.06	1.09
RB3-1		120	5.86	1000	250	31.3	321.1	40.9	39.88	1.03
RB3-2		120	5.86	1000	250	31.3	321.1	41.54	39.88	1.04
RB4-1		120	5.86	1000	250	40.0	321.1	41.43	40.61	1.02
RB4-2		120	5.86	1000	250	40.0	321.1	42.61	40.61	1.05
BSb0	[31]	150	2.94	1000	250	42.7	344.1	34.9	36.80	0.95
SVA-1	[32]	100	1.9	1400	350	81.3	282	10.83	9.94	1.09
SVA-2		100	1.9	1400	350	81.3	282	9.96	9.94	1.00
SSCA-1		100	1.9	1400	350	81.3	282	10.33	9.94	1.04
SVB-1		200	1.9	1400	350	81.3	282	42.3	48.69	0.87
SVB-2		200	1.9	1400	350	81.3	282	54.3	48.69	1.12
SSCB-1		200	1.9	1400	350	81.3	282	56.7	48.69	1.16
SB1-1	[32]	140	3	840	210	51.5	235	31.9	27.02	1.18
SB1-2		140	3	840	210	51.5	235	27.5	27.02	1.02
SB2-1		140	3	1680	420	51.5	235	29.4	28.64	1.03
SB4-1		180	3	900	225	62.6	235	37.6	43.22	0.87
SB4-2		180	3	900	225	62.6	235	43.1	43.22	1.00
SB5-1		180	3	1800	450	62.6	235	37.9	43.02	0.88
NMC-1	[33]	72	3.2	1000	333	32.5	345	10.06	10.07	1.00
NMC-2		72	3.2	1000	333	32.5	345	9.57	10.07	0.95
NMC-3		72	3.2	1000	333	32.5	345	10.4	10.07	1.03
FAC-1		72	3.2	1000	333	32.5	345	9.9	10.07	0.98
FAC-2		72	3.2	1000	333	32.5	345	10.07	10.07	1.00
FAC-3		72	3.2	1000	333	32.5	345	10.4	10.07	1.03
SB A-0	[34]	140	3.5	1200	400	33.0	300	42	41.05	1.02
SB B-0		140	3.5	1200	400	39.4	300	42.4	41.09	1.03
SB C-0		140	3.5	1200	400	49.0	300	43.4	41.55	1.04
SB D-0		140	3.5	1200	400	59.7	300	43.2	42.54	1.02
								Mean		1.026
								CV		0.072

Note: *D* represents the outer dimension of the steel tube (diameter for circular, side length for square sections), *t* denotes the thickness of the steel tube, *l*_0_ refers to the clear span, *a* is the shear span, *f*_y_ is the yield strength of the steel tube, *f*_cu_ is the concrete cube strength, *M*_ue_ is the maximum test moment, *M*_u,FE_ is the maximum FE moment, and the ratio *M*_ue_/*M*_u,FE_ represents the ratio of the maximum test moment to the maximum FE moment.

**Table 2:** Specimen details and comparison of bending moment test results with FEM results for circular CFSTs.

Specimen Label	Ref.	*D*/mm	*t*/mm	*l*_0_/mm	*a*/mm	*f*_cu_/MPa	*f*_y_/MPa	*M*_ue_ /kN·m	*M*_u,FE_/kN·m	*M*_ue_/*M*_u,FE_
CBC0-C	[35]	109.9	1	1400	300	29.25	400	7.64	7.00	1.091
CBC0-B		110.4	1.25	1400	300	29.25	400	9.08	8.60	1.056
CBC0-A		110.9	1.5	1400	300	29.25	400	11.72	10.18	1.152
CBC1		101.83	2.53	1400	300	29.25	365	11.33	10.71	1.058
CBC2		88.64	2.79	1400	300	29.25	432	10.94	10.03	1.091
CBC3		76.32	2.45	1400	300	29.25	415	6.84	6.52	1.049
CBC4		89.26	3.35	1400	300	29.25	412	11.25	11.81	0.952
CBC5		60.65	2.44	1400	300	29.25	433	3.97	3.96	1.003
CBC6		76.19	3.24	1400	300	29.25	456	9.87	8.55	1.154
CBC7		60.67	3.01	1400	300	29.25	408	5	4.68	1.068
CBC8		33.66	1.98	1400	300	29.25	442	0.93	0.97	0.955
CBC9		33.78	2.63	1400	300	29.25	460	1.2	1.26	0.950
C1	[36]	180	1.48	1200	300	64	307	25.1	22.27	1.127
C2		180	1.48	1200	300	64	307	18.8	22.27	0.844
TPB002	[37]	406	6.4	3800	1300	50	350	489	463.42	1.055
TPB003		406	6.4	3800	1300	62.5	350	489	475.30	1.029
TPB005		456	6.4	3800	1300	60	350	630	604.00	1.043
TPB006		456	6.4	3800	1300	66	350	647	620.39	1.043
BCb0	[32]	165	2.57	100	250	42.7	343.1	29.4	30.43	0.966
CVA-1	[25]	100	1.9	1400	350	81.3	282	3.5	7.156	1.284
CVA-2		100	1.9	1400	350	81.3	282	3.5	7.156	1.024
CSCA-1		100	1.9	1400	350	81.3	282	3.5	7.156	1.082
CVB-1		200	1.9	1400	350	81.3	282	1.75	33.355	0.971
CVB-2		200	1.9	1400	350	81.3	282	1.75	33.355	1.016
CSCB-1		200	1.9	1400	350	81.3	282	1.75	33.355	1.097
CB1-1		140	3	840	210	51.5	235	1.5	18.856	1.050
CB1-2		140	3	840	210	51.5	235	1.5	18.856	1.146
CB2-1		140	3	1680	420	51.5	235	3	18.625	1.154
CB4-1		180	3	900	225	62.6	235	33.9	31.18	1.087
CB4-2		180	3	900	225	62.6	235	34.9	31.18	1.119
CB5-1		180	3	1800	450	62.6	235	32.2	31.79	1.013
D1t1M20	[38]	44.45	1.25	900	300	26.84	250	0.74	0.78	0.949
D1t2M30		44.45	1.6	900	300	40.06	250	1.16	1.24	0.935
D1t3M40		44.45	2	900	300	48.32	250	1.46	1.49	0.980
D2t1M30		57.15	1.25	900	300	40.06	250	1.57	1.42	1.106
D2t2M40		57.15	1.6	900	300	48.32	250	2.03	2.11	0.962
D2t3M20		57.15	2	900	300	26.84	250	2.25	2.27	0.991
D3t1M40		63.5	1.25	900	300	48.32	250	2.03	2.08	0.976
D3t2M20		63.5	1.6	900	300	26.84	250	2.18	2.22	0.982
D3t3M30		63.5	2	900	300	40.06	250	3.16	3.19	0.991
CB A-0	[39]	89	4.5	1200	400	60.7	204	12.5	12.27	1.018
CB B-0		108	4.5	1200	400	60.7	269	18.5	19.16	0.966
CB C-0		133	4.5	1200	400	60.7	333	32.7	31.97	1.023
CB D-0		159	4.5	1200	400	60.7	333	51.5	48.60	1.060
CS	[40]	135	3	1850	620	30.2	353.3	18.5	21.89	0.845
CFST17.5	[41]	114	6.5	1000	300	49.25	245	21.8	21.11	1.033
CFST13.5		114	8.5	1000	300	49.25	245	29.78	28.95	1.029
CFST7.82		114	14.6	1000	300	49.25	245	41.53	41.97	0.990
C	[42]	165	2.7	1700	620	31.5	346	30.9	30.20	1.023
C-0L	[43]	114	2.9	1200	400	40.6	364.1	17.1	16.03	1.067
								Mean		1.033
								CV		0.076

**Table 3:** DS concrete uniaxial stress-strain curve parameters.

**Condition**	***x***	***y***	***f*** **_c(r)_** **or** ***f*** **_t(r)_**	*ε*_c(r)_ or *ε*_t(r)_	*A*_n(r)_	*B*_n(r)_	*α*_n(r)_
Compression *n* = c	*ε/ε*_c(r)_	*σ*/*f*_c(r)_	$\left(1-0.1r\right)\times 0.4{f_{cu}}^{7/6}$	$\left(1-0.15r\right)\times 291{f_{cu}}^{7/15}\times 10^{-6}$	$6.9{f_{cu}}^{-11/30}\left(1-0.15r\right)$	$1.67{\left(A_{c(r)}-1\right)}^{2}$	0.15
Tension *n* = t	*ε/ε*_t(r)_	*σ*/*f*_t(r)_	$\left(1-0.1r\right)\times 0.24{f_{cu}}^{2/3}$	$\left(1+0.1r\right)\times 33{f_{cu}}^{1/3}\times 10^{-6}$	$1.306\left(1+0.1r\right)$	$1.67{\left(A_{t(r)}-1\right)}^{2}$	0.8

**Table 4:** Concrete damage plasticity model parameters.

Parameter Name	Value
Desert sand concrete elastic modulus, *E*_c_	(1−0.1*r*)9500*f*_cu_^1/3^
Poisson’s ratio	0.2
Dilation angle	40°
Eccentricity *e*	0.1
Ratio of biaxial to uniaxial compressive strength, *f*_b0_/*f*_c0_	1.331
Viscosity parameter, *K*	0.0005

**Table 5:** Parameters for pure bending FEM of square and circular CFST members.

Section Type	*D*/mm	*L*/mm	*ρ*	*ρ*_sa_	*f*_cu_/MPa	*f*_y_/MPa	*r*
Square Circular	500	4500	0.02, 0.05, 0.08	0	30~70	235~420	0~1
			0.05	0.004~0.0245			

Note: *ρ* is the section steel ratio, where *ρ* = *A*_s_/(*A*_s +_ *A*_c_), with *A*_s_ being the steel tube area and *A*_c_ being the concrete area. *ρ*_sa_ is the section volume stirrup ratio, where *ρ*_sa_ = *v*_s_ × *f*_ys_/(*A*_s +_ *A*_c_) × *S* × *f*_y_, with *v*_s_ representing the is the volume of a single layer of stirrups, *S* being the spacing between the stirrups.

**Table 6:** Comparison between different codes for ultimate bending moment.

Ref.	Section Shape	*M*_u,Ex_/*M*_u,Eq_		*M*_u,EF_/*M*_u,Eq_		Mean	CV
		Mean	CV	Mean	CV		
This study	Square	0.982	0.089	1.003	0.047	0.993	0.068
	Circular	1.005	0.066	0.997	0.019	1.001	0.043
AIJ	Square	1.324	0.123	1.451	0.149	1.388	0.136
	Circular	1.474	0.130	1.455	0.096	1.465	0.113
AISC-LFRD	Square	1.510	0.128	1.613	0.149	1.562	0.139
	Circular	1.638	0.130	1.616	0.096	1.627	0.113
GB 50936	Square	1.090	0.117	0.991	0.058	1.041	0.088
	Circular	1.164	0.161	1.120	0.034	1.142	0.098

## Data Availability

The data presented in this study are available on request from the corresponding author due to confidentiality agreements.

## Abbreviations

The following abbreviations are used in this manuscript:

*a*	Shear span
*A*_c_	Cross-sectional area of concrete
*A*_s_	Cross-sectional area of the steel tube
AIJ	Architectural Institute of Japan
AISC	American Institute of Steel Construction
CFST	Concrete-filled steel tubular
*D*	Outer dimension of the steel tube; diameter for circular, side length for square sections
DS	Desert sand
*E*c	Elastic modulus of concert
*E*_s_	Elastic modulus of steel
(*EI*)_sc_	Steel-concrete composite flexural stiffness
FEM	Finite element model
*f*_c_	Axial compressive strength of concrete
*f*_y_	Yield strength of the steel tube
*f*_cu_	Concrete cube strength
*k*_z_	Concrete flexural stiffness reduction factor considering concrete cracking
*M*u	Ultimate bending moment
*M*_ue_	Experimental measured ultimate bending moment
*M*_u,FE_	Finite element computed ultimate bending moment
*M*_u,eq_	Formula calculated ultimate bending moment
*L*	Length of the member
*r*	Replacement ratio
*t*	thickness of the steel tube
*l*_0_	clear span
*ϕ*	Midspan curvature
ϕ	stirrup diameter
*ε*	strain
*ρ*	Steel ratio in the steel tube
*ρ*_sa_	equivalent stirrup ratios
*σ*	stress
*σ*_θ,s_	Hoop stress
*σ*_L,s_	Longitudinal stress
